# Fertility options and challenges for patients with cytogenetic infertility & disorders of sex development

**DOI:** 10.1186/1755-8166-7-S1-I20

**Published:** 2014-01-21

**Authors:** Rama Ashok Vaidya, Jaya Gogte

**Affiliations:** 1 Milan Polyclinic, 71-B Sarashwati Road, Santacruz (W), Mumbai, India

## 

Recent advances in assisted reproductive technology (ART), coupled with the emerging understanding in molecular mechanisms of disorders of sex development (DSD) and that of associated genetic infertility, have given hopes for fertility in groups of patients who till recently were denied biological parenthood.

Heterologous fertility potentiation, in the cytogenetic infertility by donor oocytes, has been successfully used by us and others. However, homologous fertility preservation for this group of patients is evolving. Cryopreservation of either the mature oocytes following ovarian stimulation in post -pubertal Turner Syndrome (TS) girls or that of ovarian tissue in prepubertal TS patients are to be applied before actual ovarian failure occurs. Single embryo-transfer and strict selection criteria and judicious use of this advanced technology are advocated to minimize maternal morbidity and mortality.

Microdissection testicular sperm extractions (Micro-TESE) in azoospermic Klinfelter syndrome patients have resulted not only in successful retrieval of spermatozoa for intracytoplasmic sperm injection (ICSI) but also in normal fertilization and clinical pregnancies. Swyer’s syndrome patients are reared as females and have female external and internal genitalia. However, with complete gonadal failure they need Oocyte donation with ICSI/IVFET for fertility. Those with partial androgen resistance (PAIS) due to genetic mutation of androgen receptor have a spectrum of abnormality like hypospadias, micropenis, undescended testes and male infertility. Though 46xy PAIS males have possibility of fatherhood complete AIS patients are female phenotype and are raised as females. This category of patients are encouraged and known to resort to fertilization of donated oocyte using husband’s sperms. Resultant embryos are transferred into surrogate uterus.

Advances in reproductive medicine in general and those in assisted reproductive technology in particular have revolutionized diagnosis of disorders of sex development and the management of the associated infertility. These advances have certainly provided fertility potentiation but require judicious application coupled with expert genetic counseling.

